# Parasternal intercostal thickening at hospital admission: a promising indicator for mechanical ventilation risk in subjects with severe COVID-19

**DOI:** 10.1007/s10877-023-00989-4

**Published:** 2023-03-24

**Authors:** Mina A. Helmy, Lydia M. Milad, Ahmed M. Hasanin, Maha Mostafa, Asser H. Mannaa, Marianne M. Youssef, Mahmoud Abdelaziz, Ramy Alkonaiesy, Mamdouh Mahmoud Elshal, Osama Hosny

**Affiliations:** 1 Department of Anesthesia and Critical Care Medicine, Cairo University, Cairo, Egypt; 2 Department of Anesthesia and Critical Care Medicine, National Cancer Institute, Cairo University, Cairo, Egypt; 3 Critical Care Medicine, Faculty of Medicine, Cairo University, Cairo, Egypt

**Keywords:** COVID-19, Parasternal intercostal thickening, Ultrasound, P_aO2_/F_IO2_ ratio, Mechanical ventilation, ROX index

## Abstract

We aimed to evaluate the ability of parasternal intercostal thickening fraction (PIC TF) to predict the need for mechanical ventilation, and survival in subjects with severe Coronavirus disease-2019 (COVID-19). This prospective observational study included adult subjects with severe COVID-19. The following data were collected within 12 h of admission: PIC TF, respiratory rate oxygenation index, ${{{\text{P}}_{{{\text{aO}}_{{\text{2}}} }} } \mathord{\left/ {\vphantom {{{\text{P}}_{{{\text{aO}}_{{\text{2}}} }} } {{\text{F}}_{{{\text{iO}}_{{\text{2}}} }} }}} \right. \kern-\nulldelimiterspace} {{\text{F}}_{{{\text{iO}}_{{\text{2}}} }} }}$ ratio, chest CT, and acute physiology and chronic health evaluation II score. The ability of PIC TF to predict the need for ventilatory support (primary outcome) and a composite of invasive mechanical ventilation and/or 30-days mortality were performed using the area under the receiver operating characteristic (AUC) analysis. Multivariate analysis was done to identify the independent predictors for the outcomes. Fifty subjects were available for the final evaluation. The AUC (95% confidence interval [CI]) for the right and left PIC TF ability to predict the need for ventilator support was 0.94 (0.83–0.99), 0.94 (0.84–0.99), respectively, with a cut off value of > 8.3% and positive predictive value of 90–100%. The AUC for the right and left PIC TF to predict invasive mechanical ventilation and/or 30 days mortality was 0.95 (0.85–0.99) and 0.90 (0.78–0.97), respectively. In the multivariate analysis, only the PIC TF was found to independently predict invasive mechanical ventilation and/or 30-days mortality. In subjects with severe COVID-19, PIC TF of 8.3% can predict the need to ventilatory support with a positive predictive value of 90–100%. PIC TF is an independent risk factor for the need for invasive mechanical ventilation and/or 30-days mortality.

**Supplementary Information:**

The online version contains supplementary material available at 10.1007/s10877-023-00989-4.

## Introduction

The Coronavirus disease-2019 (COVID-19) is a major health threat with more than 6 million deaths worldwide. A major factor in the high case-fatality is the pandemic pattern of the disease which produces a large number of critically ill patients in a short time overloading the healthcare systems. Thus, early detection of at-risk patients for deterioration and need for organ support is important during the initial evaluation of the patient [1]. It is essential to classify patients’ needs for oxygen therapy on hospital admission in order to manage health care resources properly [2, 3], and avoid the hazards of delayed initiation of invasive ventilation, which is associated with poor outcomes [4]. The ideal measure for disease severity should be accurate, non-invasive, economic, and easy for first-line physicians. Several methods for risk stratification had been reported such as computed tomography (CT) scan, clinical variables, and laboratory markers; however, none of them fulfilled the requirements of the ideal variable. There had been increased interest in ultrasonographic assessment of the activity of the diaphragm and other respiratory muscles as predictors of patient outcomes [5–7]. Ultrasound evaluation of the respiratory muscles carries several advantages, such as being a bedside tool, simple, economic and devoid of ionizing radiation [8].

Being non-invasive and easily accessible, parasternal intercostal (PIC) muscle ultrasound has gained interest as a tool for evaluating the respiratory function [9] and showed excellent accuracy in predicting non-invasive ventilation failure [5].

The aim of this work is to evaluate the ability of PIC thickening fraction PIC TF, when measured within 12 h of hospital admission to predict the need for mechanical ventilation, and survival in patients with COVID-19.

## Patients and methods

This prospective observational study was conducted in a university Hospital after the institutional research ethics board approval (N-24-2022). Written informed consent was obtained from the subject’s next of kin before enrollment. We consecutively included 50 adult subjects with severe COVID-19 (oxygen saturation [${\text{Sp}}_{{{\text{O}}_{{\text{2}}} }}$] below 94%, ${{{\text{P}}_{{{\text{aO}}_{{\text{2}}} }} } \mathord{\left/ {\vphantom {{{\text{P}}_{{{\text{aO}}_{{\text{2}}} }} } {{\text{F}}_{{{\text{iO}}_{{\text{2}}} }} }}} \right. \kern-\nulldelimiterspace} {{\text{F}}_{{{\text{iO}}_{{\text{2}}} }} }}$ ratio less than 300, and respiratory rate > 30 breaths per minute) [10]. Respiratory and hemodynamic management were carried out according to the Surviving Sepsis Campaign guidelines and our local protocols [11]. In this study, oxygen therapy was used in an escalating approach to achieve ${\text{Sp}}_{{{\text{O}}_{{\text{2}}} }} > 92\%$ and a respiratory rate < 35 breaths/min in the form of a simple mask, non-rebreathing mask, high flow nasal oxygen, non-invasive ventilation, and invasive mechanical ventilation. Subjects who required mechanical ventilation within the first 12 h were excluded from the study. Data collection included the respiratory oxygenation (ROX) index (calculated as follows: ${{{\text{Sp}}_{{{\text{O}}_{{\text{2}}} }} } \mathord{\left/ {\vphantom {{{\text{Sp}}_{{{\text{O}}_{{\text{2}}} }} } {{{{\text{F}}_{{{\text{iO}}_{{\text{2}}} }} } \mathord{\left/ {\vphantom {{{\text{F}}_{{{\text{iO}}_{{\text{2}}} }} } {{\text{respiratory}}\,{\text{rate}}}}} \right. \kern-\nulldelimiterspace} {{\text{respiratory}}\,{\text{rate}}}}}}} \right. \kern-\nulldelimiterspace} {{{{\text{F}}_{{{\text{iO}}_{{\text{2}}} }} } \mathord{\left/ {\vphantom {{{\text{F}}_{{{\text{iO}}_{{\text{2}}} }} } {{\text{respiratory}}\,{\text{rate}}}}} \right. \kern-\nulldelimiterspace} {{\text{respiratory}}\,{\text{rate}}}}}}$), ${{{\text{P}}_{{{\text{aO}}_{{\text{2}}} }} } \mathord{\left/ {\vphantom {{{\text{P}}_{{{\text{aO}}_{{\text{2}}} }} } {{\text{F}}_{{{\text{iO}}_{{\text{2}}} }} }}} \right. \kern-\nulldelimiterspace} {{\text{F}}_{{{\text{iO}}_{{\text{2}}} }} }}$ ratio, CT scan of the chest, Acute Physiology, and chronic Health Evaluation II (APACHE II) score, and laboratory markers (Ferritin, C-reactive protein, procalcitonin, D-dimer, and interleukin-6).

A radiologist, who was blinded to clinical and ultrasound information, assessed the CT scan and scored it by dividing the lung into five zones: right upper, right middle, right lower, left upper and left lower zones. Each zone received a score between 0 and 5 according to the degree of affection (Score 1 for less than 5% affection; score 2 for 5–25%; score 3 for 25–50%; score 4 for 50–75%; and score 5 for 75% or greater) [12]. An ultrasound examination of the second intercostal muscle was carried out by an expert operator (MAH who had more than 5-years’ experience in intensive care point-of-care ultrasound examination with several publications in this topic and had completed more than 50 examinations of the intercostal muscles) who had no further involvement in the study. All ultrasonographic examinations were done using the LOGIC V5 ultrasound device (GE Medical Systems Co., Ltd., China). A high-frequency linear probe (L6–12-RS, 4–16 MHz) was placed at the second intercostal space, in the parasagittal plane 3–5 cm lateral to the sternal edge while the subject was in the semi-sitting position. Respiratory phase changes were evaluated using M mode on a frozen image. The PIC TF was calculated as

$$\left ( {{{\left[ {{\text{Peak}}\,{\text{inspiratory}}\,{\text{thickness}} - {\text{end}}\,{\text{expiratory}}\,{\text{thickness}}} \right]} \mathord{\left/ {\vphantom {{\left[ {{\text{peak}}\,{\text{inspiratory}}\,{\text{thickness}} - {\text{end}}\,{\text{expiratory}}\,{\text{thickness}}} \right]} {{\text{end}}\,{\text{expiratory}}\,{\text{thickness}}}}} \right. \kern-\nulldelimiterspace} {{\text{end}}\,{\text{expiratory}}\,{\text{thickness}}}}} \right)\, \times \,{\text{100}}$$

All ultrasound assessments were done in realtime and the assessment of one side took less than one minute.

The ${{{\text{P}}_{{{\text{aO}}_{{\text{2}}} }} } \mathord{\left/ {\vphantom {{{\text{P}}_{{{\text{aO}}_{{\text{2}}} }} } {{\text{F}}_{{{\text{iO}}_{{\text{2}}} }} }}} \right. \kern-\nulldelimiterspace} {{\text{F}}_{{{\text{iO}}_{{\text{2}}} }} }}$ ratio and ROX index were evaluated in the emergency department; the CT scan was conducted immediately before ICU admission. Ultrasound examination and other data were collected after ICU admission within the first 12 h from hospital admission (all data collection was completed within 12 h of hospital admission).

The primary outcome of the study was the ability of PIC TF to predict the need for ventilatory support (non-invasive and/or invasive mechanical ventilation); other outcomes were the ability of the ROX index, ${{{\text{P}}_{{{\text{aO}}_{{\text{2}}} }} } \mathord{\left/ {\vphantom {{{\text{P}}_{{{\text{aO}}_{{\text{2}}} }} } {{\text{F}}_{{{\text{iO}}_{{\text{2}}} }} }}} \right. \kern-\nulldelimiterspace} {{\text{F}}_{{{\text{iO}}_{{\text{2}}} }} }}$ ratio, CT score and APACHE II score to predict the need for ventilatory support and the ability of PIC TF, ROX index, ${{{\text{P}}_{{{\text{aO}}_{{\text{2}}} }} } \mathord{\left/ {\vphantom {{{\text{P}}_{{{\text{aO}}_{{\text{2}}} }} } {{\text{F}}_{{{\text{iO}}_{{\text{2}}} }} }}} \right. \kern-\nulldelimiterspace} {{\text{F}}_{{{\text{iO}}_{{\text{2}}} }} }}$ ratio, CT score and APACHE II score to predict a composite of invasive mechanical ventilation and/or 30-days mortality.

## Sample size

MedCalc Software version 14 (MedCalc Software bvba, Ostend, Belgium) was used to calculate an area under receiver operating characteristic curve (AUC) of 0.75 and to achieve study power and alpha error of 80% and 0.05, respectively. We assumed that ventilatory support would be required in 35% of the cases. A total of 46 subjects were calculated, with at least 16 subjects requiring ventilatory support.

### Statistical analysis

The statistical package for social science (SPSS) for Microsoft version 26 (IBM Corp., Armonk, NY, USA) and MedCalc software were used for statistical analyses of the raw data. The included cohort were divided according to the need for ventilatory as well as according to the need for invasive mechanical ventilation and/or 30-days mortality. The Kolmogorov-Smirnov test was used to detect normally distributed variables. Normally distributed data were represented as mean ± standard deviation, and skewed data were represented as median (quartiles). The unpaired t-test was used for the comparison of normally distributed data, and the Mann-Whitney test was used to analyze skewed data. The Chi-square was used to analyze nominal data. The AUC was calculated to analyze the ability of each of PIC TF, APACHE II score, ROX index, ${{{\text{P}}_{{{\text{aO}}_{{\text{2}}} }} } \mathord{\left/ {\vphantom {{{\text{P}}_{{{\text{aO}}_{{\text{2}}} }} } {{\text{F}}_{{{\text{iO}}_{{\text{2}}} }} }}} \right. \kern-\nulldelimiterspace} {{\text{F}}_{{{\text{iO}}_{{\text{2}}} }} }}$ ratio, and CT score to predict study outcomes. The Youden index was then identified to detect the best cut-off value and a comparison of ROC curves, using the Delong et al. approach, was conducted. A multivariate logistic regression model was used to identify the independent risk factors for the study outcomes and their odds ratio (OR), and 95% confident interval (CI) were calculated. The mean PIC TF was calculated to be included in the multivariate analysis instead of the right and left PIC TF. P-value < 0.05 was considered statistically significant for all the data.

## Results

**Fig. 1:**
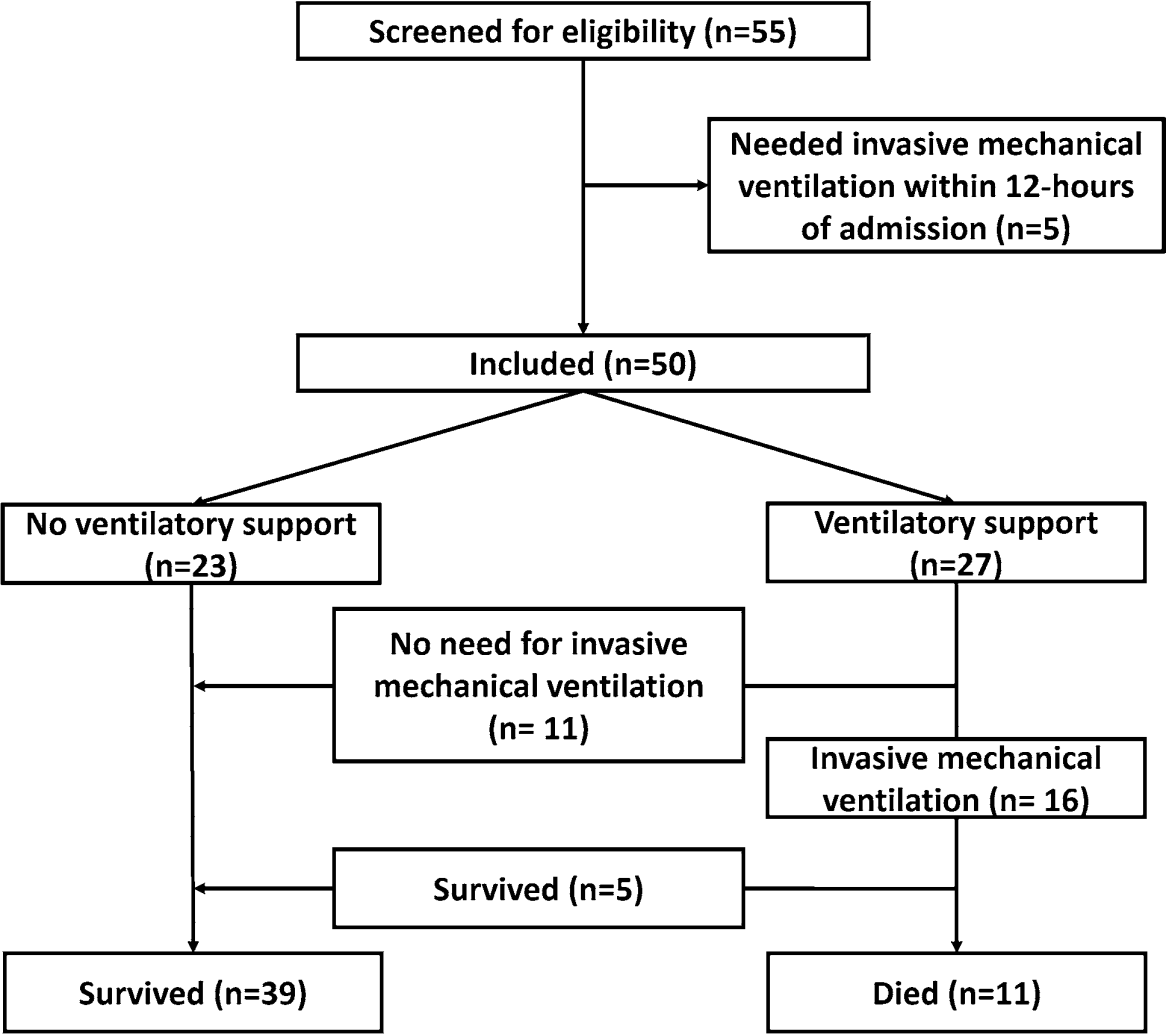
Subjects’ enrollment and outcomes

Fifty-five subjects were screened for eligibility, five subjects were excluded as they received mechanical ventilation within 12 h of hospital admission and fifty subjects were included and were available for final evaluation. Twenty-three/fifty (46%) subjects required ventilatory support, 16/50 (32%) subjects required invasive mechanical ventilation and 11/50 (22%) died. (Fig. 1)

Subjects’ characteristics are presented in Table 1. Patients’ characteristics according to the need for ventilatory support and according to the composite of need for invasive mechanical ventilation and/or 30 days mortality are presented in Supplementary files (S1 and S2).

**Table 1:** Demographic, clinical, laboratory, and ultrasound data. Data are presented as mean ± standard deviation, median (quartiles), and frequency (%)

	All (n = 50)
Age (years)	54.8 ± 14.4
Male sex	27 (54%)
Body mass index (kg/m^2^)	26.2 (23.4, 27.8)
Comorbidity (%)	
Hypertension	27 (54%)
Ischemic heart disease	5 (10%)
Atrial fibrillation	2 (4%)
Stroke	1 (2%)
Bronchial asthma	1 (2%)
Diabetes mellitus	14 (28%)
Chronic kidney disease	6 (12%)
${{{\text{P}}_{{{\text{aO}}_{{\text{2}}} }} } \mathord{\left/ {\vphantom {{{\text{P}}_{{{\text{aO}}_{{\text{2}}} }} } {{\text{F}}_{{{\text{iO}}_{{\text{2}}} }} }}} \right. \kern-\nulldelimiterspace} {{\text{F}}_{{{\text{iO}}_{{\text{2}}} }} }}$ ratio	216 ± 47
APACHE II score	10 (7, 14)
ROX index	13.8 ± 4.3
Rt-PIC TF (%)	7.2 (4.7, 16.3)
Lt-PIC TF (%)	6.7 (3.2, 15.5)
M-PIC TF (%)	7 (4.1, 14.8)
CT score	11.8 ± 4.5
Ferritin (pg/mL)	495 (273, 1044)
C-reactive protein (mg/L)	100 (36, 143)
Procalcitonin (mcg/L)	0.19 (0.1, 0.38)
D-dimer (mcg/mL)	1.2 (1.6, 3.0)
Interleukin-6 (pg/mL)	106 (45, 247)

*APACHE II* Acute Physiology Assessment and Chronic Health Evaluation II, *CT* computed tomography, *Lt-PIC TF* left parasternal intercostal thickening fraction, *M-PIC TF* mean parasternal intercostal thickening fraction, *PaO*_2_/*FiO*_2_ ratio of arterial oxygen partial pressure to fractional inspired oxygen, *ROX* respiratory rate oxygenation, *Rt-PIC TF* right parasternal intercostal thickening fraction

The PIC TF was higher in patients needing ventilatory support and in the composite outcome of need for invasive mechanical ventilation and/or 30-days mortality (supplementary files: S1 and 2).

In the univariate analysis, the PIC TF, APACHE II, P_aO2_/F_iO2_ ratio, ROX index, and CT score were identified as the risk factors for each of need for ventilatory support and need for invasive mechanical ventilation and/or 30-days mortality. (Table 2) In the multivariate analysis, only the PIC TF was found to independently predict invasive mechanical ventilation and/or 30-days mortality (OR [95% CI] 1.28 [1.04–1.57], P-value: 0.018) and was likely to predict the need for ventilatory support (OR [95% CI] 1.43 [0.96–2.14], P-value: 0.082) (Table 3).

**Table 2:** Univariate analysis for the need for ventilatory support and composite outcome of invasive mechanical ventilation and /or 30 days mortality

	Need for ventilatory support		Need for invasive mechanical ventilation and/or 30 days mortality	
	Odd ratio (95% CI)	P value	Odd ratio (95% CI)	P value
Age	1.04 (1-1.09)	0.06	1.03 (0.98–1.07)	0.268
Male sex	2.34 (0.75–7.37)	0.145	2.48 (0.71–8.67)	0.156
BMI	1.06 (0.97–1.17)	0.225	1.12 (0.99–1.26)	0.066
APACHE II	1.18 (1.04–1.35)	0.012	1.15 (1.02–1.29)	0.023
Diabetes	1.19 (0.34–4.14)	0.781	1.25 (0.32–4.83)	0.746
Hypertension	0.60 (0.19–1.84)	0.370	0.60 (0.18–2.02)	0.410
IHD	0.53 (0.08–3.51)	0.513	0.27 (0.04–1.81)	0.178
Atrial fibrillation	6.40 (0.15–275.62)	> 0.999	11.89 (0.27–515.42)	> 0.999
Stroke	2.67 (0.03–256.40)	> 0.999	1.48 (0.01–143.19)	> 0.999
CKD	0.14 (0.02–1.29)	0.082	1.25 (0.32–4.83)	0.746
Bronchial asthma	3.64 (0.04–344.66)	> 0.999	6.59 (0.07–623.92)	> 0.999
${{{\text{P}}_{{{\text{aO}}_{{\text{2}}} }} } \mathord{\left/ {\vphantom {{{\text{P}}_{{{\text{aO}}_{{\text{2}}} }} } {{\text{F}}_{{{\text{iO}}_{{\text{2}}} }} }}} \right. \kern-\nulldelimiterspace} {{\text{F}}_{{{\text{iO}}_{{\text{2}}} }} }}$ ratio	0.96 (0.93–0.98)	< 0.001	0.97 (0.95–0.99)	0.002
ROX	0.48 (0.33–0.70)	< 0.001	0.67 (0.53–0.84)	0.001
Rt-PIC TF	1.54 (1.18-2.00)	0.001	1.41 (1.16–1.71)	0.001
Lt-PIC TF	1.89 (1.24–2.90)	0.003	1.27 (1.11–1.45)	0.001
M-PIC TF	1.97 (1.32–2.95)	0.001	1.36 (1.14–1.62)	0.001
CT score	1.64 (1.24–2.17)	0.001	1.42 (1.15–1.75)	0.001
Ferritin	1.001 (1.00-1.002)	0.060	1.00 (1.00–1.00)	0.255
C-reactive protein	1.00 (0.99–1.01)	0.920	1.00 (0.99–1.01)	0.396
Procalcitonin	4.10 (0.63–26.85)	0.141	6.57 (0.80-54.14)	0.080
D-dimer	1.12 (0.82–1.54)	0.488	0.98 (0.70–1.36)	0.888
Interleukin-6	1.00 (1.00–1.00)	0.502	1.00 (1.00–1.00)	0.508

*APACHE II* Acute Physiology and Chronic Health Evaluation II, *BMI* body mass index, *CKD* chronic kidney disease, *CT* computed tomography, *IHD* ischemic heart disease, *Lt-PIC TF* left parasternal intercostal thickening fraction, *M-PIC TF* mean parasternal intercostal thickening fraction, *ROX* respiratory rate oxygenation, *Rt-PIC TF* right parasternal intercostal thickening fraction

**Table 3:** Multivariate analysis for the need for ventilation and composite outcome of invasive mechanical ventilation and/or 30 days mortality

	Need for ventilatory support		Need for invasive mechanical ventilation and/or 30 days mortality	
	Odd ratio (95% CI)	P value	Odd ratio (95% CI)	P value
M-PIC TF	1.43 (0.96–2.14)	0.082	1.28 (1.04–1.57)	0.018
APACHE II	0.94 (0.75–1.17)	0.562	1.09 (0.91–1.29)	0.353
${{{\text{P}}_{{{\text{aO}}_{{\text{2}}} }} } \mathord{\left/ {\vphantom {{{\text{P}}_{{{\text{aO}}_{{\text{2}}} }} } {{\text{F}}_{{{\text{iO}}_{{\text{2}}} }} }}} \right. \kern-\nulldelimiterspace} {{\text{F}}_{{{\text{iO}}_{{\text{2}}} }} }}$ ratio	0.97 (0.93–1.02)	0.193	0.99 (0.96–1.02)	0.537
ROX	0.79 (0.40–1.56)	0.492	1.02 (0.65–1.61)	0.921
CT score	1.43 (0.75–2.71)	0.686	1.23 (0.87–1.73)	0.244

*APACHE II* Acute Physiology and Chronic Health Evaluation II, *CT* computed tomography, *CI* confidence interval, *M-PIC TF* mean parasternal intercostal thickening fraction, *ROX* respiratory rate oxygenation

**Fig. 2:**
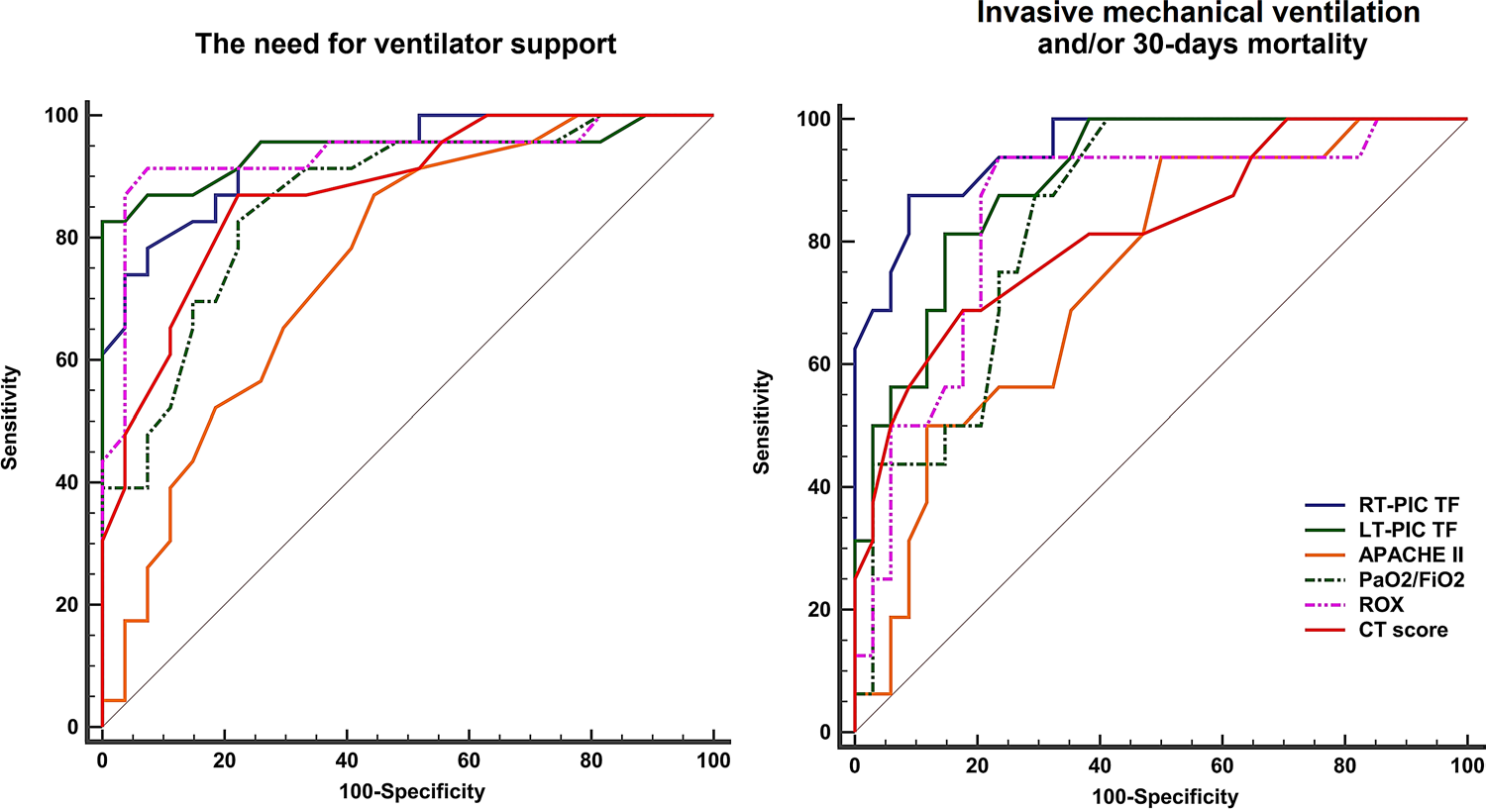
The receiver operating characteristic curve for the ability to predict the need for mechanical ventilation (to the left) and to predict invasive mechanical ventilation and/or 30-days mortality (to the right). *APACHE II* Acute Physiology and Chronic Health Evaluation II, *AUC* area under receiver operating characteristic curve, *CT* computed tomography, *Lt-PIC TF* left parasternal intercostal thickening fraction, ${{{\text{P}}_{{{\text{aO}}_{{\text{2}}} }} } \mathord{\left/ {\vphantom {{{\text{P}}_{{{\text{aO}}_{{\text{2}}} }} } {{\text{F}}_{{{\text{iO}}_{{\text{2}}} }} }}} \right. \kern-\nulldelimiterspace} {{\text{F}}_{{{\text{iO}}_{{\text{2}}} }} }}$: ratio of arterial oxygen partial pressure to fractional inspired oxygen, *ROX* respiratory rate oxygenation, *Rt-PIC TF* right parasternal intercostal thickening fraction

The AUC for PIC TF ability to predict the need for ventilator support was 0.94 (0.83–0.99), 0.94 (0.84–0.99) for the right and left side, respectively, with a cut-off value of > 8.3%. The AUCs for the PIC TF and ROX were higher than that of the APACHE II. (Table 4; Fig. 2)

**Table 4:** The AUC analysis for the ability to predict the need for ventilator support and composite of invasive mechanical ventilation and/or 30-day mortality

	AUC (95% CI)	Sensitivity % (95% CI)	Specificity% (95% CI)	PPV% (95% CI)	NPV% (95% CI)	Cut-off value
The need for ventilator support						
Rt-PIC TF	0.94 (0.83–0.99)*	78 (56–93)	93 (76–99)	90 (70–97)	83 (70–92)	> 8.3%
Lt-PIC TF	0.94 (0.84–0.99)*	83 (61–95)	100 (87–100)	100 (82–100)	87 (74–94)	> 8.3%
ROX index	0.93 (0.82–0.98)*	91 (72–99)	93 (76–99)	91 (73–98)	93 (77–98)	< 13
${{{\text{P}}_{{{\text{aO}}_{{\text{2}}} }} } \mathord{\left/ {\vphantom {{{\text{P}}_{{{\text{aO}}_{{\text{2}}} }} } {{\text{F}}_{{{\text{iO}}_{{\text{2}}} }} }}} \right. \kern-\nulldelimiterspace} {{\text{F}}_{{{\text{iO}}_{{\text{2}}} }} }}$ ratio	0.86 (0.73–0.94)	83 (61–95)	78 (58–91)	76 (60–69)	84 (68–93)	≤ 219
CT score	0.88 (0.75–0.95)	87 (66–97)	78 (58–91)	77 (62–87)	88 (71–95)	> 11
APACHE II	0.76 (0.62–0.87)	87 (66–97)	56 (35–75)	63 (52–72)	83 (62–94)	> 8
Invasive mechanical ventilation and/or 30-day mortality						
Rt-PIC TF	0.95 (0.85–0.99)*^†^	88 (62–98)	91 (76–98)	82 (61–93)	94 (81–98)	> 11.4%
Lt-PIC TF	0.90 (0.78–0.97)	82 (54–96)	86 (69–95)	72 (53–86)	91 (76–96)	> 9.7%
ROX index	0.85 (0.72–0.93)	94 (70–100)	77 (56–89)	65 (50–78)	96 (79–100)	< 13
${{{\text{P}}_{{{\text{aO}}_{{\text{2}}} }} } \mathord{\left/ {\vphantom {{{\text{P}}_{{{\text{aO}}_{{\text{2}}} }} } {{\text{F}}_{{{\text{iO}}_{{\text{2}}} }} }}} \right. \kern-\nulldelimiterspace} {{\text{F}}_{{{\text{iO}}_{{\text{2}}} }} }}$ ratio	0.84 (0.71–0.93)	100 (79–100)	59 (41–75)	53 (43–63)	100 (83–100)	≤ 229
CT score	0.82 (0.68–0.91)	69 (41–89)	82 (66–93)	65 (45–80)	85 (73–92)	> 13
APACHE II	0.74 (0.60–0.86)	94 (70–100)	50 (32–68)	47 (38–56)	94 (71–99)	> 8

*APACHE II* Acute Physiology and Chronic Health Evaluation II, *AUC* area under receiver operating characteristic curve, *CI* confidence interval, *CT* computed tomography, *Lt-PIC TF* left parasternal intercostal thickening fraction, *NPV* negative predictive value, *PPV* positive predictive value, *ROX* respiratory rate oxygenation, *Rt-PIC TF* right parasternal intercostal thickening fraction

*Significance in relation to APACHE II score

^†^Significance in relation to CT score

The AUC for the Rt-PIC TF to predict invasive mechanical ventilation and/or 30 days mortality (AUC [95% CI] 0.95 [0.85–0.99]) was higher than that for the APACHE II score (AUC [95% CI] 0.74 [0.60–0.86]) and CT score (AUC [95% CI] 0.82 [0.68–0.91]) (P-value = 0.007, and 0.043, respectively). (Table 4; Fig. 2)

## Discussion

We report that PIC TF evaluated within 12 h of admission can accurately predict the need for ventilator support and composite outcome of invasive mechanical ventilation and/or 30-day mortality in subjects with severe COVID 19. Furthermore, the PIC TF was the only independent predictor of invasive mechanical ventilation and/or 30-day mortality among other risk tools, namely the APACHE II score, ROX index, ${{{\text{P}}_{{{\text{aO}}_{{\text{2}}} }} } \mathord{\left/ {\vphantom {{{\text{P}}_{{{\text{aO}}_{{\text{2}}} }} } {{\text{F}}_{{{\text{iO}}_{{\text{2}}} }} }}} \right. \kern-\nulldelimiterspace} {{\text{F}}_{{{\text{iO}}_{{\text{2}}} }} }}$ ratio and CT score. The intercostal muscles are among the accessory muscles of respiration and their activity increase in case of increased work of breathing [9]. Electromyographic and sonographic studies showed inverse relationship between the PIC TF function and diaphragmatic function in non-COVID-19 patients with respiratory failure [9, 13]. Patients with COVID-19 have diaphragmatic dysfunction which was explained by possible viral invasion and/or fibrotic changes in the diaphragm [14]. This diaphragmatic dysfunction showed clear association with the need for ventilatory support as well as in-hospital mortality in patients with severe COVID-19 [6, 7].

The increased intercostal muscle activity, as a compensatory mechanism to the reduced diaphragmatic activity [9], lead to the interest in the use of the PIC TF as a simple surrogate to the diaphragmatic excursion. The PIC TF was previously found to be able to predict failure of non-invasive ventilation in patients with COVID-19 with comparable accuracy to diaphragmatic excursion and the advantage of being easier [5]. In this study we report a novel application of intercostal muscle ultrasound as an accurate marker of severity in the early phases of the disease and interestingly, we found that the cut-off values of the PIC TF were close to our previous study [5]. Furthermore, the PIC TF values in subjects who had unfavorable outcome in this study (median [quartiles]: 16[11, 27]% for need for ventilatory support and 22[13, 29]% for the composite outcome) are close to that reported by Dres et al. in non-COVID-19 patients with failed spontaneous breathing trial (median [quartiles]: 18 [10, 33]%) [9].

Ultrasound assessment of the intercostal muscles is characterized by being simple and accessible; hence, it could be an ideal tool for triaging and risk stratification. PIC TF showed better accuracy than other clinical and radiological variables such as the CT score and APACHE score which adds to its bedside and non-invasive nature. Furthermore, PIC TF was the only independent predictor for the composite outcome of invasive mechanical ventilation and/or 30-day mortality.

### Limitations

Our study has some limitations; It is a single-center study and all ultrasound examinations were performed by a single operator; however, previous reports showed that the inter-rater agreement for the PIC TF were acceptable [9]; therefore, future research is recommended to verify our cut-off values. Second, we evaluated the parasternal intercostal muscles only in the second space since a previous physiologic study reported a descending reduction in PIC thickening from the 2nd to 5th intercostal spaces [15]. Similarly, in other clinical studies, the second space was used to reflect PIC muscle activity [9, 13].

## Conclusion

In subjects with severe COVID-19, PIC TF evaluated within 12 h of admission could be a useful triaging tool in predicting the need for mechanical ventilation. A PIC TF of 8.3% can predict the need to ventilation support with a positive predictive value of 90–100%. PIC TF is an independent risk factor for the need for invasive mechanical ventilation and/or 30 days mortality.

## Supplementary Information

Below is the link to the electronic supplementary material.

Supplementary material 1 (DOCX 18.4 kb)

## Data Availability

The data that support the findings of this study are available from the authors upon reasonable request after permission of Cairo university.
