# Supplementary material for: Parasternal intercostal thickening at hospital admission: a promising indicator for mechanical ventilation risk in subjects with severe COVID-19
Source: J Clin Monit Comput. 2023 Mar 24;37(5):1287–93. doi: 10.1007/s10877-023-00989-4 (PMC10037364; doi:10.1007/s10877-023-00989-4)
Supplement: Supplementary file 1 — Supplementary material 1 (DOCX 18.4 kb) [file 10877_2023_989_MOESM1_ESM.docx]

S 1: Patients’ characteristics according to the need for ventilatory support. Data are presented as mean ±standard deviation, median (quartiles), and frequency (%)

|  | Need for ventilatory support | |
| --- | --- | --- |
|  | No (n= 27) | Yes (n= 23) |
| Age (years) | 51 ±15 | 59 ±13 |
| Male sex | 15 (56%) | 8 (35%) |
| Body mass index (kg/m^2^) | 26 (23, 28) | 26 (23, 29) |
| P_aO2_/F_iO2_ ratio | 243 ±32 | 186 ±43 |
| APACHE II score | 8 (6, 12) | 13 (10, 17) |
| ROX index | 17 ±3 | 10 ±3 |
| Rt-PIC TF (%) | 5 (2.9, 6.7) | 16.7 (9.1, 25.0) |
| Lt-PIC TF (%) | 4.1 (2.8, 6.3) | 15.6 (11.7, 21.6) |
| M-PIC TF (%) | 4.4 (3.4, 6.1) | 15.8 (11.2, 26.9) |
| CT score | 9 ±3 | 15 ±4 |
| Ferritin (pg/mL) | 394 (225, 895) | 604 (414, 1240) |
| C-reactive protein (mg/L) | 104 (25, 136) | 86 (43, 154) |
| Procalcitonin (mcg/L) | 0.17 (0.08, 0.38) | 0.19 (0.12, 0.52) |
| D-dimer (mcg/mL) | 1.8 (1.6, 2.9) | 2.2 (1.5, 3.2) |
| Interleukin-6 (pg/mL) | 144 (49, 247) | 66 (34, 247) |

APACHE II: Acute Physiologic Assessment and Chronic Health Evaluation II, CT: computed tomography, Lt-PIC TF: left parasternal intercostal thickening fraction, M-PIC TF: mean parasternal intercostal thickening fraction, PaO_2_/FiO_2_: ratio of arterial oxygen partial pressure to fractional inspired oxygen, ROX: respiratory rate oxygenation, Rt-PIC TF: right parasternal intercostal thickening fraction.

S 2: Patients’ characteristics according to the composite outcome of the need for invasive mechanical ventilation and/or 30 days mortality. Data are presented as mean ±standard deviation, median (quartiles), and frequency (%)

|  | Invasive mechanical ventilation and/or 30 days mortality | |
| --- | --- | --- |
|  | No (n= 34) | Yes (n= 16) |
| Age (years) | 53 ±15 | 58 ±13 |
| Male sex | 18 (53%) | 5 (31%) |
| Body mass index (kg/m^2^) | 26 (23, 28) | 26 (23, 35) |
| P_aO2_/F_iO2_ ratio | 234 ±41 | 180 ±39 |
| APACHE II score | 9 (7, 12) | 14 (10, 17) |
| ROX index | 15 ±4 | 10 ±3 |
| Rt-PIC TF (%) | 5.4 (3.1, 7.6) | 23.4 (14.6, 25.6) |
| Lt-PIC TF (%) | 5.3 (2.9, 7.3) | 17.7 (12.1, 29.3) |
| M-PIC TF (%) | 4.9 (3.8, 8.4) | 22.1 (13.0, 29.3) |
| CT score | 10 ±3 | 15 ±5 |
| Ferritin (pg/mL) | 414 (242, 946) | 724 (364, 1242) |
| C-reactive protein (mg/L) | 109 (25, 154) | 78 (52, 139) |
| Procalcitonin (mcg/L) | 0.19 (0.10, 0.36) | 0.17 (0.12, 0.80) |
| D-dimer (mcg/mL) | 1.8 (1.6, 2.8) | 2.7 (1.4, 3.6) |
| Interleukin-6 (pg/mL) | 160 (74, 285) | 60 (26, 106) |

APACHE II: Acute Physiologic Assessment and Chronic Health Evaluation II, CT: computed tomography, Lt-PIC TF: left parasternal intercostal thickening fraction, M-PIC TF: mean parasternal intercostal thickening fraction, PaO_2_/FiO_2_: ratio of arterial oxygen partial pressure to fractional inspired oxygen, ROX: respiratory rate oxygenation, Rt-PIC TF: right parasternal intercostal thickening fraction.
